# Suicide ideation, planning, and attempts: the case of the Latinx LGB youth

**DOI:** 10.15171/hpp.2019.28

**Published:** 2019-08-06

**Authors:** Javier F. Boyas, Tatiana Villarreal-Otálora, Luis R. Alvarez-Hernandez, Mariam Fatehi

**Affiliations:** University of Georgia, School of Social Work, 279 Williams St., Athens, GA, 30602, USA

**Keywords:** Suicide, Latinos, LGB Persons, Adolescence

## Abstract

**Background: ** Guided by an ecological systems theory (EST) framework, the purpose of the present study was to investigate how multiple micro, mezzo, and macro factors influence the suicidality continuum from suicidal ideation to suicide attempt among Latinx LGB (lesbian, gay, and bisexual) youth living in the United States.

**Methods:** Data for this cross sectional-study included 451 participants who self-identified as Latinx LGB on the 2017-National Youth Risk Behavioral Survey. The analysis explored micro, mezzo, and macro-level factors’ association with three suicidality outcomes (ideation, planning, and attempt) at the bivariate and multivariate level. Since the outcome variables were dichotomized, univariate logistic regressions and backward elimination logistic regressions were used.

**Results:** The most commonly reported suicidal behavior was ideation (n = 173; 40%), followed by planning (n = 150; 34%), and then attempt (n = 64; 21%). Findings from the backward elimination logistic regression on suicidal ideation suggest the best set of independent variables are being bullied at school (odds ratio [OR] = 2.81; CI: 1.61–4.89), experiencing sexual assault(OR = 2.32; CI: 1.32–4.07), experiencing depressive symptoms (OR = 1.99; CI: 1.07– 3.69),being cannabis use (OR = 1.76; CI: 1.08–2.89), and being female (OR = 1.72; CI: 1.01–2.93).For suicide planning the model suggested, experiencing depressive symptoms (OR = 3.21; CI:1.74–5.91), cannabis use (OR = 2.46; CI: 1.49–4.07), being bullied at school (OR = 2.04; CI:1.17–3.58), and experiencing sexual assault (OR = 1.88; CI: 1.07–3.31) exhibited the strongest relationships. Suicide attempt was significantly associated with cannabis use (OR = 3.12; CI:1.60–6.08), experiencing depression (OR= 2.89; CI: 1.30–6.43), experiencing sexual assault (OR = 2.77; CI: 1.34–5.71), and being bullied at school (OR = 2.34; CI: 1.12–4.91).

**Conclusion: ** Given the findings of this study, it is essential that tailored suicide prevention efforts be established that uniquely address the intersections of race/ethnicity and sexual orientation and how this intersection influences micro, mezzo, and macro factors associated with suicide ideation, planning, and attempt among Latinx LGB adolescents.

## Introduction

Despite the recognition that Latinx and sexual minority youth are groups that are at elevated risk of suicidal behaviors in the United States, little attention has been given to the experiences of Latinx youth who identify as lesbian, gay, and bisexual (LGB). This research oversight is sobering given that, separately, both groups are considered high-risk because they have some of the highest incidence rates of suicide. In 2017, a higher number of Latinx youth (8.2%) reported attempting suicide compared to non-Hispanic whites (6.1%).^1^ Moreover, in 2017, 16.4% of Latinx youth seriously considered attempting suicide, 13.5% made a suicide plan, and 8.2% engaged in lethal action. A meta-analysis focused on sexual minority youth suggests that LGB youth are at increased risk of attempted suicide compared to heterosexual youth.^2^ The same study revealed that relative to Latinx heterosexual youth, Latinx homosexual youth were 3.71 more likely to have attempted suicide, while bisexual youths were 3.69 more likely to have attempted suicide. Thus, suicide has now become one of the greatest threats to the health of Latinx LGB youth during the healthiest period of the lifespan.^3^ For the purpose of this study, Latinx is a gender-neutral term describing youth living in the United States whose nationality group or the country in which the person or her/his parent’s or descendants were born in is a Latin American country.^4,5^

Using the ecological systems theory (EST),^6^ the purpose of the present study was to investigate how multiple micro, mezzo, and macro factors influence the evolution from suicidal ideation to suicide attempt among Latinx LGB youth. EST is a valuable paradigm because of the recognition of the strong association between individuals and various elements that make up their environments. EST underscores that a person’s development and worldview is shaped by the transactional relationship with their social milieu.^6^ According to the available literature, Latinx and LGB youth suffer from a number of micro-level stressors associated with suicidality. These micro-level stressors include cannabis use,^7-9^ and experiencing: intimate partner violence,^10,11^ sexual assault,^10-13^ and depressive symptoms.^14-17^ At the micro-level, sex differences have also been found in relation to suicide, with females experiencing higher levels of ideation than males.^18-20^ At the mezzo level, identifying as a Latinx youth and having an LGB identity are risk factors in school settings. One study suggests that an association exists between identifying as a Latinx youth, experiencing bullying, and suicidal attempts.^21^ Another study suggests that70% of youth reported being bullied at school because of their sexual orientation, with 43% having been bullied on school property.^22^ At the macro level, understanding the landscape of immigration-related policies are necessary since several studies suggest that immigration policies and the related exclusionary climate adversely affects the mental health of Latinx youth.^23-25^

It is critical that more research is developed that focuses on investigating which socio-ecological factors promote increased notions of suicide among Latinx LGB youth because this group has some of the most elevated risks for suicide, which appears to disproportionately burden this group. To deal with this ethnic disparity, it is important to determine the pathways contributing to increased risk. Such knowledge is essential for developing a better understanding of the etiology of suicide among Latinx LGB youth, furthering risk assessment, and designing tailored evidence-based suicide prevention programs that address the particular needs of this population.

The following research questions guided the present study:

1. What micro factors—such as depressive symptoms, cannabis use, IPV, and sexual assault—are associated with suicidality (i.e., suicide ideation, planning, and attempt) among Latinx LGB youth?
2. What mezzo factors—such as experiences at school with bullying— are associated with suicidality (i.e., suicide ideation, planning, and attempt) among Latinx youth?
3. Are macrosystem factors—such as state-level immigration-policy climate—associated with suicidality (i.e., suicide ideation, planning, and attempt) among Latinx LGB youth?

## Material and Methods

### Study design and sample

This secondary data analysis used cross-sectional data from the 2017 National Youth Risk Behavioral Survey (YRBS). Using a three-stage cluster sampling design, the CDC has administered the YRBS annually since 1990 to monitor the health of 9th through 12th-grade students living in the United States. Data extracted for this cross-sectional study represents a subsection of 2017 YRBS participants who self-identified as Latinx (N = 3653) and that also identified as LGB (N = 451). All demographic variables on the YRBS are weighted.

### Instrumentation 

The focus of this study was on parceling out suicidal behavior (i.e., ideation, planning, and attempt) among Latinx LGB youth. Thus, suicidal thoughts and behaviors were collapsed into three dependent variables: suicidal ideation; suicide planning; and suicide attempt. All three single-item variables were measured using responses to the following questions on the YRBS: During the last 12 months… “did you ever seriously consider attempting suicide?”; “did you make a plan about how you would attempt suicide?”; and, “how many times did you actually attempt suicide?”. The latter was dichotomized into yes/no response categories. If the participant responded that they carried out 1, or more, suicide attempts, they were coded as “yes”. All response chooses were coded as 0 = no and 1 = yes.

*EST domains*

To represent the micro, mezzo, and macro-level domains, six EST variables were included in the analysis: *cannabis use*, *depressive symptoms*, *intimate partner violence* (IPV), *sexual assault*, *bullied at school*, and *immigration policy climate*. Cannabis use, depressive symptoms, IPV, sexual assault, and bullied at school were based on the participants’ answers to the following YRBS questions: During the last 12 months… “how many times did you use marijuana?”; “did you feel so sad or hopeless almost every day for two weeks or more in a row that you stopped doing some usual activities?”; “how many times did someone you were dating or going out with physically hurt you on purpose?”; “how many times did anyone force you to do sexual things that you did not want to do?”; and, “have you ever been bullied on school property?”. The response categories for marijuana use, depressive symptoms, sexual assault, and bullied at school were dichotomized into no (e.g., no endorsement of depressive symptoms or no reported incident of sexual assault) and yes (e.g., yes depressive symptoms were endorsed or yes reported at least one incident of sexual assault). Responses were coded as follows: 0 = no, and 1 = yes. For IPV, the response categories were collapsed into three categories: 0 = no IPV, 1 = yes IPV, and 2 = has never dated. Immigration climate was created using the participant’s state of residency and Hatzenbuehler and colleagues’^25^ 14-item state policy index. The policy index assigns a score representing the states immigration policy climate ranging from 1 (*most inclusionary*) to 5 (*most exclusionary*). We collapsed their categories into three: 1 = inclusionary, 2 = neither inclusionary nor exclusionary, and 3 = exclusionary. Lastly, participant’s *age* (12 - 14, 15, 16, 17, and ≥18) and* sex* (0 = male versus 1 = female) were also included in the analysis.

### Statistical analysis 

Univariate statistics were used to examine the variables’ distributions and missingness, which revealed a large percentage of missingness in two variables, suicide attempt (31%) and sexual orientation (24%). This missingness was handled by first determining the nature of missingness using Little’s MCAR test.^26^ The statistically significant findings (*P* ≤ .001) from Little’s MCAR test suggested that data was not missing-at-random; thus, commonly used imputation techniques were not appropriate to use, and instead, listwise deletion was used. The rest of the variables’ missingness was below the acceptable percentage of 10%. After addressing missingness, a series of binary logistic regressions were conducted to explore each variables relationship to the three dependent variables (i.e., ideation, planning, and attempt) and the independent variables. A prior significance threshold of .25 or less was set for inclusion in the final multivariate analyses.^27^ This final analysis included three multiple logistic regression models using backward elimination to find the set of best EST factors that of suicidality. In order to evaluate the models, four properties were examined: Akaike’s information criterion (AIC), Bayesian information criterion (BIC), *R*^2^ adjusted (_ADJ_), and Mallow’s *C*_p_. The optimal model selected was one that exhibited: the smallest AIC, BIC, and Mallow’s *C*_p_ values; and, the largest *R*^2^_ADJ_ value.^28^ For the final multivariate analyses, statistical significance was measured at the 95% confidence interval level (*P* ≤ 0.05). All analyses were conducted using STATA version 12 (College Station, TX).^29^ Odd ratios (OR) and 95% confidence intervals (CI) were reported for the bivariate and multivariate results.

## Results

### Participant general characteristics

Table 1 shows the descriptive statistics of the variables used in the analysis. The Latinx LGB youth in our sample was predominantly female (n = 292) and 74% identified as bisexual with most youth between the age of 15 to 17 (n = 328). Many of the respondents reported not using cannabis (n = 275). Twenty-one percent of respondents reported experiencing depressive symptoms, while another 22% reported experiencing sexual assault. The majority of the sample also reported experiencing IPV (n = 237). Most of the respondents reported living in inclusionary states (n = 311).

**Table 1:** Demographic and suicidality characteristics of participants

**Variable**	**No.**	**%**
Sex		
Male	156	34.82
Female	292	65.42
Orientation		
Gay or Lesbian	88	25.81
Bisexual	253	74.19
Age		
12 to 14	77	17.07
15	109	24.17
16	104	23.06
17	115	25.50
18 and older	46	10.20
Cannabis use		
Yes	129	31.93
No	275	68.07
Depressive symptoms		
Yes	92	21.20
No	342	78.80
Intimate partner violence		
Yes	237	57.25
No	51	12.32
Never dated	126	30.43
Sexual assault		
Yes	93	22.25
No	325	77.75
Bullied at school		
Yes	117	26.53
No	324	73.47
Immigration climate		
Inclusionary	311	71.82
Neither	99	22.86
Exclusionary	23	5.31
Suicidal ideation		
Yes	173	39.77
No	262	60.23
Suicide planning		
Yes	150	34.25
No	288	65.75
Suicide attempt		
Yes	64	20.58
No	247	79.42

*N* = 451 (numbers may not add up to 100% due to missing values or rounding off thus weighted percentages are reported).

Nearly 40% of our sample reported experiencing suicide ideation, 34% reported making suicide plans, and almost 21% attempted suicide. Out of the 173 Latinx LGB youth who endorsed suicidal ideation in our sample, 71% of them also created a suicide plan and 34% of them attempted suicide. Similarly, of the 150 respondents who reported suicide planning 34% of them also reported having attempted suicide. All of the 64 Latinx LGB youth who reported having previously attempted suicide also reported a history of suicide ideation.

### Predictors of suicidality variables among Latinx adolescents: univariate analysis 

Results of logistical regressions showed that several micro and mezzo factors were significantly associated with each of the suicidality variables among Latinx LGB youth (see Table 2). Significant sex differences emerged with regards to suicide ideation, but not planning or attempt (OR: 1.22, 95% CI: .80-1.87; OR: .99, 95% CI: .54-1.81, respectively). Latinx LGB females had 1.69 higher odds of suicide ideation compared to Latinx LGB males. Significant age differences also emerged in to relation to suicide ideation and planning, but not attempts. Latinx LGB youth between the ages of 12-14 had .65 higher odds of suicide ideation than adolescents who were 15 years of age, while adolescents 16 years of age had .57 higher odds of reporting suicide ideation. However, Latinx LGB youth who were 16 years of age had .66 lower odds of suicidal planning than those in their early adolescence.

**Table 2:** Bivariate analysis of micro, mezzo, and macro-level variables for suicidality

**Variable**	**Suicidal Ideation**	**Suicide Planning**	**Suicide Attempt**
	**OR (95% CI)**	**OR (95% CI)**	**OR (95% CI)**
Sex			
Male	Ref	Ref	Ref
Female	1.69 (1.11–2.57)*	1.22 (0.80–1.87)	0.99 (0.54–1.81)
Sexual orientation			
Gay or Lesbian	Ref	Ref	Ref
Bisexual	0.91 (0.55–1.5)	0.86 (0.52–1.43)	1.40 (0.71–2.73)
Age			
12 to 14	Ref	Ref	Ref
15	0.65 (0.35–1.18)*	0.69 (0.37–1.29)	1.08 (0.46–2.58)
16	0.57 (0.31–1.06)*	0.66 (0.35–1.25)*	0 .67 (2.67–1.69)
17	0.73 (0.40–1.33)	0.81 (0.44–1.49)	1.08 (0.46–2.53)
18 and older	0.64 (0.30–1.37)	0.97 (0.46–2.08)	1.07 (0.38–2.98)
Cannabis use			
No	Ref	Ref	Ref
Yes	2.12 (1.37–3.28)*	2.80 (1.79–4.38)*	3.14 (1.75–5.62)*
Depressive symptoms			
No	Ref	Ref	Ref
Yes	3.67 (2.25–5.91)*	5.45 (3.32–8.93)*	5.25 (2.81–9.83)*
Intimate partner violence			
No	Ref	Ref	Ref
Yes	4.49 (2.24–8.98)*	4.87 (2.52–9.43)*	8.56 (3.78–9.39)*
Never dated	0 .83 (0.52–1.31)	0.84 (0.52–1.36)	050 (0.23–1.11)
Sexual assault			
No	Ref	Ref	Ref
Yes	3.12 (1.91–5.08)*	3.01 (1.86–4.88)*	3.78 (1.99–7.14)*
Bullied at school			
No	Ref	Ref	Ref
Yes	3.23 (2.07–5.03)*	3.61 (2.31–5.62)*	3.80 (2.14–6.76)*
Immigration climate			
Inclusionary	Ref	Ref	Ref
Neither	0.78 (0.48–1.25)	0.86 (0.53–1.40)	1.22 (0.66–2.26)
Exclusionary	0.91 (0.38–2.18)	0.62 (0.24–1.62)	0 .92 (0.29–2.88)

N = 451, * indicates significance of *P* ≤ 0.2. Ref = indicates the reference or comparison group; OR = Odds ratios; 95% CI = confidence intervals for odds.

Among Latinx LGB youth, cannabis use, experiencing depressive symptoms, experiencing IPV, experiencing sexual assault and being bullied at school were all significantly associated with suicide ideation, planning, and attempt. Latinx LGB cannabis users had 2.12 higher odds of suicide ideation, 2.80 higher odds of planning, and 3.14 higher odds of attempting suicide than Latinx LGB non-users. Latinx LGB youth who experienced depressive symptoms had 3.67 higher odds of suicide ideation, 5.45 higher odds of planning, and 5.25 higher odds of attempting suicide than Latinx LGB with no reported depressive symptoms. Latinx LGB youth who reported having experienced sexual assault had 3.12 higher odds of suicide ideation, 3.01 higher odds of planning, and 3.78 higher odds of attempting suicide than Latinx LGB who reported not having experienced sexual assault. Latinx LGB youth who experienced being bullied at school were 3.23 times more likely to report suicide ideation, 3.61 time more likely to plan suicide, and 3.80 time more likely to attempt suicide than Latinx LGB who reported not experiencing bullying. In terms of IPV, Latinx LGB youth who reported engaging in non-violent intimate partner relationships were 4.49 more likely to report suicide ideation, 4.87 times more likely to plan suicide, but 8.56 time more likely to attempt suicide as compared to those who reported never experiencing IPV. Youth’s specific sexual orientation (i.e., gay and lesbian versus bisexual) and the state-level immigration climate did not share a significant relationship with suicide ideation, planning, or attempt.

### Predictors of suicidality variables among Latinx adolescents: multivariate analysis 

*Suicidal ideation*

The backward logistic regression model examining suicidal ideation among Latinx LGB youth suggested an optimal model that included five EST variables: sex, cannabis use, depressive symptoms, experiencing sexual assault, and being bullied at school (see Table 3). This model explained 11.67% of the variance in suicidal ideation outcomes among Latinx LGB. Latinx LGB females were nearly twice as more likely to report suicidal ideation as their male counterparts. Latinx LGB adolescents who experienced depressive symptoms were 1.99 more times likely to report suicide ideation compared to adolescents who had not experienced depressive symptoms, while adolescents who experienced sexual assault were 2.32 more likely to report suicide ideation compared to adolescents who had not experienced sexual assault. Latinx adolescents who reported cannabis use were 1.76 more likely to report suicide ideation compared to adolescents who were not cannabis users. The highest odds of reporting suicide ideation was among Latinx LGB youth who experienced bullying at school. They were almost three times more likely to report suicidal ideation as compared to those who reported no history of experiencing bullying.

*Suicide planning*

Findings based on the backwards logistic regression suggests the best set of independent variables of suicide planning among Latinx LGB youth included: cannabis use, depressive symptoms, experiencing sexual assault, and being bullied at school (see Table 3). This model with four EST factors explained 12.94% of the variance in suicidal planning. The Latinx youth in our sample who reported being bullied at school were 2.04 times more likely to report suicidal planning than those that reported not being bullied at school. Similarly, Latinx LGB youth who reported cannabis use were 2.46 times more likely to engage in suicide planning than those who reported no cannabis use. Latinx LGB youth experienced sexual assault were 1.88 times more likely to engage in suicide planning than those who did not experience sexual assault. The greatest odds of suicidal planning were amongst respondents who experienced depressive symptoms. Latinx youth who had depressive symptoms were three times more likely than those who reported no depressive symptoms to have made a suicide plan.

**Table 3:** Best subset models on suicide ideation, planning and attempt

**Variable**	**Suicidal Ideation**	**Suicide Planning**	**Suicide Attempt**
	**OR (95% CI)**	**OR (95% CI)**	**OR (95% CI)**
Gender			
Male	Ref	-	-
Female	1.72 (1.01-2.93)*	-	-
Cannabis use			
No	Ref	Ref	Ref
Yes	1.76 (1.08-2.89)*	2.46 (1.49-4.07)***	3.12 (1.60-6.08)***
Depressive symptoms			
No	Ref	Ref	Ref
Yes	1.99 (1.07-3.69)*	3.21 (1.74-5.91)***	2.89 (1.30-6.43)**
Intimate partner violence			
No	-	-	-
Yes	-	-	-
Never dated	-	-	-
Sexual assault			
No	Ref	Ref	Ref
Yes	2.32 (1.32-4.07)**	1.88 (1.07-3.31)*	2.77 (1.34-5.71)**
Bullied at school			
No	Ref	Ref	Ref
Yes	2.81 (1.61-4.89)***	2.04 (1.17-3.58)**	2.34 (1.12-4.91)*
*R* ^ 2 ^_ADJ_	0.14	0.15	0.17

N = 451, *P ≤ 0.05, **P ≤ 0.01, ***P ≤ 0.001. Ref = indicates the reference or comparison group; OR = Odds ratios; 95% CI = confidence intervals for odds; ADJ = adjusted.

*Suicide attempt*

Results from the backward logistic regression examining suicide attempt explained the most variance out of the three suicidality outcome variables. Specifically, the backward elimination process suggests the same four EST factors as the suicide planning model be included: cannabis use, depressive symptoms, experiencing sexual assault, and being bullied at school. This model with four EST factors explained 16.88% if the variance in suicide attempt and correctly classified 83.57% of the cases. Latinx LGB youth who had depressive symptoms were 2.89 times more likely to have attempted suicide than those with no depressive symptoms. Similarly, Latinx youth who experienced sexual assault were 2.77 times more likely to attempt suicide than those who did not experience sexual assault. Latinx LGB youth who were bullied at school had 2.34 times higher odds of attempting suicide than those who were not bullied. Respondents who used cannabis had the highest odds of attempting suicide; they were 3.12 times more likely to attempt suicide as compared to those who did not use cannabis.

## Discussion

The results indicate that suicidality progress along the continuum from ideation to planning to attempt is a non-linear process among this population. Nearly half of the Latinx LGB youth who reported suicidal ideation did not report attempting suicide. More specifically, the results show that nearly 40% of Latinx LGB reported suicide ideation, another 34% developed a suicide plan, and nearly 21% attempted suicide. The prevalence rates of suicidal thoughts and behaviors reported in this study are higher than those reported by both groups independently in other studies.^1^ These findings underscore the need for suicidality research to increase its focus on youth who hold multiple collective identities. The shared identities that intersect race/ethnicity and sexual orientation likely create a unique set of stressful circumstances that are driven by the system interaction of oppression, domination, and discrimination.^30^ This systemic intersection constructs a social milieu that is constricting, often resulting in detrimental experiences for marginalized groups, such as Latinx LGB youth. Such context is important for health and mental health professionals to remember given that the development of an individual’s identity and the capacity to function in everyday life is contingent on sociocultural and environmental processes.^31^

Consistent with existing studies, suicide ideation among Latinx LGB youth was significantly associated with sex,^18-20^ and experiencing depressive symptoms,^14-17^ sexual assault,^10-13^ and being bullied at school.^21,22^ The findings corroborate other studies suggesting that significant sex differences exist with regards to suicide ideation, but not planning or attempt.^32^ The findings show that the sex paradox of suicide behavior also exist among Latinx LGB youth. However, it is still unknown as to whether these gender differences are related to stigma in reporting ideation among boys, or because it is more socially acceptable and feminine for girls to be more cognizant of their emotional distress.^33^ More specifically, research should explore the relationship between the multiple identities of ethnicity, sexual orientation, sex and gender.

Consistent with existing studies,^21,22,34-36^ the results indicate that being bullied at school is one of the consistent significant correlates of suicided ideation, planning, and attempt among Latinx LGB. Moreover, the results suggest that being bullied at school was the strongest factor associated with suicide ideation. These findings are very important given that school environments play a significant role in terms of development among youth that reaches far beyond academics, in large part due to their relationship with peers. The relationships in which they engage, both positive and negative, influence their mental health and social development.^37^ Regrettably, for Latinx LGB youth, school is an unsafe space where they encounter damaging and degrading messages about not only their ethnic group, but also about their sexual orientation.^35^ Considering the percentage of Latinx LGB youth who lack support from family and friends,^35^ the degrading and damaging messages heard in school place Latinx LGB youth in a vulnerable position. It is often these experiences that propel Latinx LGB youth to develop feelings of insecurity, rejection, lack of self-esteem, depression, psychosomatic complaints, and feelings of shame associated with their identity.^38^

Similar to other studies,^14-17^ the results suggest that experiencing a mood disorder such as depressive symptoms is significantly associated with suicide ideation, planning, and attempt. This result is not surprising given that several of the harmful events experienced by Latinx LGB youth, such as lacking family support and acceptance, having unsupportive peer relationships, and experiencing negative social interactions often contribute to feelings of depression.^35,39^ This finding is noteworthy considering that Latinx youth are disproportionately more likely to suffer from depressive symptoms than other racial and ethnic groups,^40,41^ but they are also 53% less likely to have ever received mental health care^41^ and they are less likely to receive treatment consistent with recommended guidelines.^42^ As a result, mental health issues among Latinx youth often go overlooked and untreated.

Among Latinx LGB adolescents, the results of this study corroborate other studies suggesting that cannabis use is significantly associated with suicide ideation, planning, and attempt.^7-9^ Moreover, in the present study, cannabis use was the strongest correlate of suicide attempt. This relationship may suggest a number of possibilities. It could imply that cannabis use impairs psychological and emotional functioning among Latinx LGB youth, which may bring about self-destructive actions that have tragic consequences, such as attempting suicide. However, this finding could also imply that cannabis use among Latinx LGB adolescents is a way of managing suicide thoughts and behaviors.^43^ This explanation gives further credence to the self-medication hypothesis, which asserts that individuals use psychoactive drugs to lessen harmful emotional or cognitive states, such as suicide ideation, planning, and attempt.^44^

### Implications

Based on the results, several implications can be drawn. First, health professionals should recognize that factors that induce suicide ideation, planning, and attempt vary among Latinx LGB. For example, in some instances, Latinx LGB youth attempted suicide, but never ideated or planned it. Thus, each sequence of the suicide continuum should be treated independently. Second, depressive symptoms directly influenced most of the suicide stages, except for suicide attempt. Depressive symptoms appear to take a psychological toll on Latinx LGB youth, which may bring about more negative thoughts. Professionals who interface with Latinx LGB should take an inventory of their level of depressive symptoms. If depressive symptoms are high, practitioners should work to develop culturally-responsive intervention plans that include strategies to lessen depression but also promote psychological resilience. Similarly, health professionals should monitor levels of cannabis use among Latinx LGB youth since they were significantly associated with suicide ideation, planning, and risk and because Latinx youth in middle school are almost twice more likely to report cannabis use compared to non-Latinx White youth,^45^ and LGB adolescents (50.4%) are more likely than non-LGB adolescents (28.8%) to report cannabis use.^46^ This will be even more important today since more states in the United States have legalized recreational use of cannabis. Third, it is common for Latinx LGB youth to seek informal assistance from family, friends, and significant others but not formal providers.^47^ Although this practice may serve youth well, this inclination should also be complemented by increasing professional outreach to this population. However, it should be noted that outreach efforts may not reach their full potential because of a shortage of diversity in the health and mental health workforce in the United States and a lack of contextually, culturally, and linguistically appropriate programs. There is growing recognition that identifying LGB counselors of color or programs that relate directly to their experiences with LGBTQ- and race-based discrimination is challenging.^22^ Thus, initiatives that support cultivating a more inclusive workforce and programs that focus on the intersection of race/ethnicity and sexual orientation are especially needed. Fourth, there is a clear need to develop and implement anti-bullying federal and state policies protecting Latinx LGB students in schools. As of 2018, only 19 states and the District of Columbia have fully enumerated anti-bullying laws.^48^ This suggests that more than half of the US does not protect Latinx LGB youth in schools from harassment, discrimination, and bigotry based on sexual orientation and/or gender identity.

### Limitations

Despite the contributions made by the current study, several limitations should be acknowledged. First, the cross-sectional research design does not allow for any causal inferences. Second, the majority of the measures were single-items. Third, the variable “suicide attempt” had an unusually large percentage of missing data. Fourth, the dataset only included information about sexual identity (being gay, lesbian, and bisexual) but not other forms of sexual behavior or sexual attraction, which are other dimensions of sexual orientation.^49^ Data on gender identity was limited. For example, Latinx youth were not given the options of self-identifying as queer, nor could they identify as transgender, or other non-binary gender identities. Last, the Latinx population is not a monolithic community; however, the diversity within this group was not captured by the analysis. There are several nationalities under the umbrella term of “Latinx”. Unfortunately, the dataset did not allow for examining within-group differences that can highlight some of the differences that exist within this broad group. The dataset also did not account for differentiating between youth who were born in the United States versus those that had migrated to the United States.

## Conclusion

Data from this study suggest that suicide ideation, planning, and attempt are highly prevalent among Latinx LGB adolescents. As a result, suicide has now become one of the single greatest health threats to a subset of the Latinx population, the largest racial/ethnic minority group in the United States. The results reveal that factors associated with each stage of suicidality vary significantly among Latinx LGB adolescents, which partially explains why the strongest factor associated with each model of suicidality differed. Overall, the independent factors examined in the present study did not account for a large variance in each regression model. Thus, using the EST framework to explain suicidality for this population is helpful, but more research is needed that explores situational and cultural factors of Latinx LGB youth and how these contextual and cultural experiences shape the suicide spectrum. Additionally, a better understanding and increasing awareness of the intersections of race/ethnicity and sexual orientation and how this intersection influence suicidality may provide a pathway to lessen the burden of suicide Latinx LGB adolescents. The present study had a majority female sample, which highlights the need to develop further research that takes into account the experiences of cisgender Latinx males who are sexual minorities. Given the findings, it is essential that tailored suicide prevention campaigns be established that uniquely address micro, mezzo, and macro factors associated with suicide ideation, planning, and attempt among Latinx LGB adolescents. This is a call to action for families, peer groups, researchers, and practitioners to engage in the creation of novel interventions and messaging efforts that target this highly vulnerable group.

## Ethical approval

This secondary data analysis was approved by the University of Georgia’s ethics committee.

## Competing interests

The authors declare that they have no competing interests.

## Funding

No external funding was obtained for the current study.

## Authors’ contributions

JFB developed the concept and design of this research project; developed the manuscript; carried out the analysis and interpretation; developed the manuscript, and approved the final version of the manuscript. TVO participated in the data analysis, data interpretation and writing of the manuscript. LRAH contributed to the literature review and development of the discussion section. MF contributed to the literature review and development of the discussion section. All four authors have reviewed, approved, and consented to the submission, and they are accountable for all aspects of its accuracy and integrity in accordance with ICMJE criteria.

## Acknowledgments

The authors would like to thank the many Latinx LGB youth who contributed to the NSDUH survey. We would also like to thank reviewers for their thoughtful and useful comments/critiques. As a result, we believe the quality of the manuscript was enhanced.

## References

[1] Centers for Disease Control and Prevention. Surveillance summaries: Youth risk behavior surveillance—United States, 2017 (MMWR Surveillance Summaries 67 No. SS-8). 2018. Available from: https://www.cdc.gov/mmwr/volumes/67/ss/ss6708a1.htm. Accessed 11 December 2018.

[2] Di Giacomo E, Krausz M, Colmegna F, Aspesi F, Clerici M (2018). Estimating the risk of attempted suicide among sexual minority youths: a systematic review and meta-analysis. JAMA Pediatr.

[3] Birnbaum AS, Linver MR. Adolescent physical development and health. In: Creasey GL, Jarvis PA, eds. Adolescent Development and School Achievement in Urban Communities: Resilience in the Neighborhood. New York, NY: Routledge; 2013. p. 53-64.

[4] Gamio A. Latinx: A Brief Guidebook. Princeton LGBTQ Center. 2016. Available from: https://www.academia.edu/29657615/Latinx_A_Brief_Guidebook. Accessed 7 June 2019.

[5] Hayes-Bautista DE, Chapa G (1987). Latino terminology: conceptual bases for standardized terminology. Am J Public Health.

[6] Bronfenbrenner U (1979). Ecological systems theory. Annals of Child Development.

[7] Delforterie MJ, Lynskey MT, Huizink AC, Creemers HE, Grant JD, Few LR (2015). The relationship between cannabis involvement and suicidal thoughts and behaviors. Drug Alcohol Depend.

[8] Pompili M, Serafini G, Innamorati M, Biondi M, Siracusano A, Di Giannantonio M (2012). Substance abuse and suicide risk among adolescents. Eur Arch Psychiatry Clin Neurosci.

[9] Borges G, Benjet C, Orozco R, Medina-Mora ME, Menendez D (2017). Alcohol, cannabis and other drugs and subsequent suicide ideation and attempt among young Mexicans. J Psychiatr Res.

[10] Olshen E, McVeigh KH, Wunsch-Hitzig RA, Rickert VI (2007). Dating violence, sexual assault, and suicide attempts among urban teenagers. Arch Pediatr Adolesc Med.

[11] Bouris A, Everett BG, Heath RD, Elsaesser CE, Neilands TB (2016). Effects of victimization and violence on suicidal ideation and behaviors among sexual minority and heterosexual adolescents. LGBT Health.

[12] Dube SR, Anda RF, Felitti VJ, Chapman DP, Williamson DF, Giles WH (2001). Childhood abuse, household dysfunction, and the risk of attempted suicide throughout the life span: findings from the Adverse Childhood Experiences Study. JAMA.

[13] Molnar BE, Berkman LF, Buka SL (2001). Psychopathology, childhood sexual abuse and other childhood adversities: relative links to subsequent suicidal behavior in the US. Psychol Med.

[14] Peña JB, Kuerbis A, Lee R, Herman D (2018). Risk profiles for suicide attempts, drug use, and violence among Dominican, Mexican, Puerto Rican, and Non-Hispanic White youth in New York City: implications for suicide prevention initiatives. Centro J.

[15] Benjet C, Menendez D, Albor Y, Borges G, Orozco R, Medina-Mora ME (2018). Adolescent predictors of incidence and persistence of suicide-related outcomes in young adulthood: a longitudinal study of Mexican youth. Suicide Life Threat Behav.

[16] Cardoso JB, Szlyk HS, Goldbach J, Swank P, Zvolensky MJ (2018). General and ethnic-biased bullying among Latino students: exploring risks of depression, suicidal ideation, and substance use. J Immigr Minor Health.

[17] Romero AJ, Edwards LM, Bauman S, Ritter MK. Risk factors for Latina adolescents’ mental health and well-being. In: Preventing Adolescent Depression and Suicide Among Latinas: Resilience Research and Theory. Cham: Springer; 2014. p. 35-46.

[18] Gunn III JF, Goldstein SE, Gager CT (2018). A longitudinal examination of social connectedness and suicidal thoughts and behaviors among adolescents. Child Adolesc Ment Health.

[19] Rew L, Young C, Brown A, Rancour S (2016). Suicide ideation and life events in a sample of rural adolescents. Arch Psychiatr Nurs.

[20] Centers for Disease Control and Prevention. Surveillance summaries: Youth risk behavior surveillance—United States, 2017 (MMWR Surveillance Summaries 67 No. SS-8). 2018. Available from: https://www.cdc.gov/mmwr/volumes/67/ss/ss6708a1.htm. Accessed 11 December 2018.

[21] Romero AJ, Bauman S, Borgstrom M, Kim SE (2018). Examining suicidality, bullying, and gun carrying among Latina/o youth over 10 years. Am J Orthopsychiatry.

[22] Human Rights Campaign. LGBTQ Youth Report. 2018. Available from: https://assets2.hrc.org/files/assets/resources/2018-YouthReport-NoVid.pdf?_ga=2.224988050.980811362.1546535175-184302017.1546535175. Accessed 1 January 2019.

[23] Gulbas LE, Zayas LH, Yoon H, Szlyk H, Aguilar-Gaxiola S, Natera G (2016). A mixed-method study exploring depression in US citizen-children in Mexican immigrant families. Child Care Health Dev.

[24] Lovato K, Lopez C, Karimli L, Abrams LS (2018). The impact of deportation-related family separations on the well-being of Latinx children and youth: a review of the literature. Child Youth Serv Rev.

[25] Hatzenbuehler ML, Prins SJ, Flake M, Philbin M, Frazer MS, Hagen D (2017). Immigration policies and mental health morbidity among Latinos: a state-level analysis. Soc Sci Med.

[26] Osterlind SJ, Tabachnick BG, Fidell LS (2001). SPSS for window workbook to accompany: using multivariate statistics.

[27] Bursac Z, Gauss CH, Williams DK, Hosmer DW (2008). Purposeful selection of variables in logistic regression. Source Code Biol Med.

[28] Lindsey C, Sheather S (2011). Variable selection in linear regression. Stata J.

[29] StataCorp. Stata Statistical Software: Release 15. College Station, TX: StataCorp LLC; 2017.

[30] Cyrus K (2017). Multiple minorities as multiply marginalized: Applying the minority stress theory to LGBTQ people of color. J Gay Lesbian Ment Health.

[31] Duarté-Vélez YM, Bernal G (2007). Suicide behavior among Latino and Latina adolescents: conceptual and methodological issues. Death Stud.

[32] Reyes-Portillo JA, Lake AM, Kleinman M, Gould MS (2019). The relationship between descriptive norms, suicide ideation, and suicide attempts among adolescents. Suicide Life Threat Behav.

[33] Canetto SS, Sakinofsky I (1998). The gender paradox in suicide. Suicide Life Threat Behav.

[34] Mueller AS, James W, Abrutyn S, Levin ML (2015). Suicide ideation and bullying among US adolescents: examining the intersections of sexual orientation, gender, and race/ethnicity. Am J Public Health.

[35] Human Rights Campaign. Supporting and caring for our Latino LGBT youth. 2013. Available from: https://assets2.hrc.org/files/assets/resources/LatinoYouthReport-FINAL.pdf. Accessed 13 December 2018.

[36] Duong J, Bradshaw C (2014). Associations between bullying and engaging in aggressive and suicidal behaviors among sexual minority youth: the moderating role of connectedness. J Sch Health.

[37] Marin P, Brown B. The school environment and adolescent well-being: Beyond academics, child trends: research brief. 2008. Available from: http://www.childtrends.org/Files/Child_Trends-2008_11_14_RB_SchoolEnviron.pdf. Accessed 13 December 2018.

[38] Shetgiri R (2013). Bullying and victimization among children. Adv Pediatr.

[39] Hall WJ (2018). Psychosocial risk and protective factors for depression among lesbian, gay, bisexual, and queer youth: a systematic review. J Homosexuality.

[40] Lechner WV, Janssen T, Kahler CW, Audrain-McGovern J, Leventhal AM (2017). Bi-directional associations of electronic and combustible cigarette use onset patterns with depressive symptoms in adolescents. Prev Med.

[41] Merikangas KR, Burnstein M, Swendsen J, Avenevoli S, Case B, Georgiades K (2011). Service utilization for lifetime mental disorder in U.S. adolescents: results from the National Comorbidity Survey-Adolescent Supplement (NCS-A). J Am Acad Child Adolsc.

[42] Caetano R, Kaplan MS, Huguet N, McFarland BH, Conner K, Giesbrecht N (2013). Acute alcohol intoxication and suicide among United States ethnic/racial groups: findings from the National Violent Death Reporting System. Alcohol Clin Exp Res.

[43] Newcomb MD, Vargas-Carmona J, Galaif ER (1999). Drug problems and psychological distress among a community sample of adults: Predictors, consequences, or confound?. J Community Psychol.

[44] Khantzian EJ (1997). The self-medication hypothesis of substance use disorders: a reconsideration and recent applications. Harv Rev Psychiatry.

[45] Johnston LD, Miech RA, O’Malley PM, Bachman JG, Schulenberg JE. Demographic subgroup trends among adolescents in the use of various licit and illicit drugs, 1975–2017 (Monitoring the Future Occasional Paper No. 90). Ann Arbor, MI: Institute for Social Research, University of Michigan; 2018. Available from: http://www.monitoringthefuture.org/pubs/occpapers/mtf-occ90.pdf. Accessed 1 June 2019.

[46] Kann L, McManus T, Harris WA, Shanklin SL, Flint KH, Queen B (2018). Youth Risk Behavior Surveillance—United States, 2017. MMWR Surveill Summ.

[47] Birkett M, Newcomb ME, Mustanski B (2015). Does it get better? A longitudinal analysis of psychological distress and victimization in lesbian, gay, bisexual, transgender, and questioning youth. J Adolesc Health.

[48] Human Rights Watch. Like walking through a hailstorm: Discrimination against LGBT youth in US schools. 2016 Available from: https://www.hrw.org/report/2016/12/07/walking-through-hailstorm/discrimination-against-lgbt-youth-us-schools#. Accessed 20 December 2018.

[49] Saewyc E, Bauer G, Skay C, Bearinger L, Resnick M, Reis E. Measuring sexual orientation in adolescent health surveys: evaluation of eight school-based surveys. J Adolesc Health 2004;35(4):345.e1-15. 10.1016/j.jadohealth.2004.06.002 15830439

